# Supplementary material for: Structural and Functional Differences in the Gut and Lung Microbiota of Pregnant Pomona Leaf-Nosed Bats
Source: Microorganisms. 2025 Aug 13;13(8):1887. doi: 10.3390/microorganisms13081887 (PMC12388707; doi:10.3390/microorganisms13081887)
Supplement: Supplementary file 1 [file microorganisms-13-01887-s001.zip › Figure S1, S2.pdf]

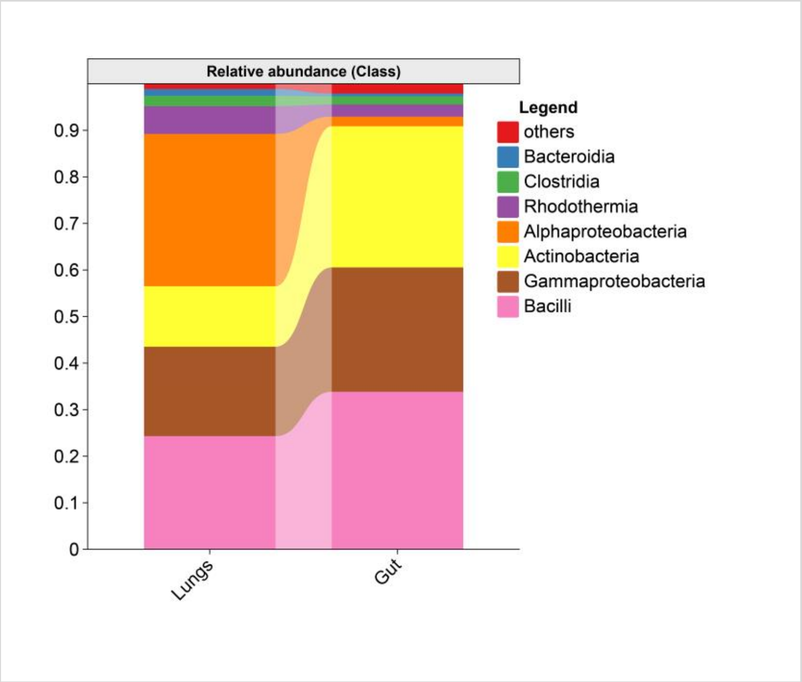

Figure S1. Bacterial classes Bacilli, Gammaproteobacteria, and Actinobacteria were more abundant in the guts; however, Alphaproteobacteria, Rhodothermia, and Bacteroidia were more abundant in the lungs.

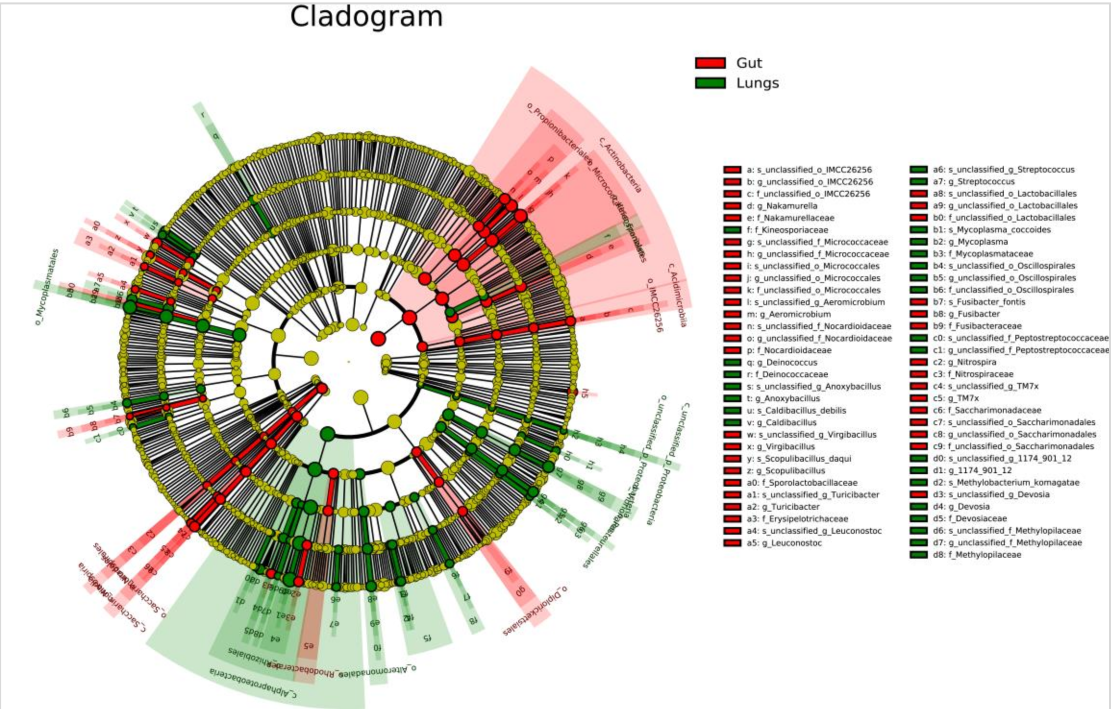

Figure S2. LEfSe cladogram results confirmed the microbial abundances and phylogenetic relationships of the detected taxa in the two anatomical sites.
