# Supplementary material for: Structural and Functional Differences in the Gut and Lung Microbiota of Pregnant Pomona Leaf-Nosed Bats
Source: Microorganisms. 2025 Aug 13;13(8):1887. doi: 10.3390/microorganisms13081887 (PMC12388707; doi:10.3390/microorganisms13081887)
Supplement: Supplementary file 1 [file microorganisms-13-01887-s001.zip › Table S1, 3,4,5,6,7,8,9.pdf]

Suppl. Table S1. Microbial sequence quality scores and GC% of guts and lung samples from nine pregnant Pomona leaf-nosed bats

Sample ID #	Total raw reads	Total clean reads	Total clean tags	Q20 (%)	Q30 (%)	GC (%)
Preg.L.1	122,846	122,553	117,289	99.1	96.4	52.2
Preg.L.2	120,578	119,662	106,742	98.9	95.9	53.1
Preg.L.3	94,467	93,867	78,182	98.8	95.6	51
Preg.L.4	122,307	121,469	109,227	98.7	95.3	52.1
Preg.L.5	120,205	119,926	113,446	98.8	95.6	54.9
Preg.L.6	92,862	92,311	84,215	98.8	95.7	55.3
Preg.L.7	127,837	127,140	117,721	98.7	95.5	55.4
Preg.L.8	112,528	111,875	103,567	98.9	95.8	53
Preg.L.9	123,098	122,644	114,723	98.8	95.7	55
Preg.G.1	131,068	130,584	121,810	98.8	95.5	54.7
Preg.G.2	128,595	128,293	121,595	99	96.2	56.6
Preg.G.3	124,903	124,473	110,377	98.9	95.9	55.5
Preg.G.4	124,199	123,888	116,692	98.8	95.7	56.7
Preg.G.5	125,335	125,000	117,928	98.9	96	56.9
Preg.G.6	121,987	121,563	114,209	98.8	95.7	56.8
Preg.G.7	131,911	131,270	120,982	98	93.2	52.3
Preg.G.8	131,703	131,143	115,112	98.8	95.5	55.7
Preg.G.9	130,820	130,367	115,664	98.8	95.6	56.6
Total	2,187,249 (avg. 121513.83)	2,178,028 (avg. 121,001.56)	1,999,481 (avg. 111,082.28)	98.8 (avg.)	95.6 (avg.)	54.66 (avg.)

Suppl. Table S3. Information on the relative dominant taxa in both groups.

**OTU**	**Lungs**	**Guts**
ASV_1	0.19019	0.10493
ASV_2	0.00636	0.26200
ASV_3	0.05529	0.08055
ASV_4	0.11560	0.00161
ASV_5	0.09173	0.00681
ASV_6	0.06637	0.02903
ASV_7	0.01823	0.05356
ASV_8	0.00048	0.07370
ASV_9	0.04546	0.01212
ASV_10	0.01913	0.03181
ASV_11	0.00173	0.02385
ASV_12	0.00877	0.01260
ASV_13	0.00904	0.01065
ASV_14	0.01196	0.00725
ASV_15	0.01446	0.00386
ASV_16	0.01091	0.00683
ASV_17	0.01232	0.00417

ASV_18	0.01130	0.00528
ASV_19	0.01119	0.00455
ASV_20	0.00856	0.00656
**Phylum**		
Proteobacteria	0.51927	0.28772
Firmicutes	0.26561	0.35630
Actinobacteriota	0.13009	0.30586
Bacteroidota	0.07429	0.03217
Others	0.01064	0.01786
**Class**		
Bacilli	0.24326	0.33848
Gammaproteobacteria	0.19204	0.26711
Actinobacteria	0.12979	0.30323
Alphaproteobacteria	0.32719	0.02060
Rhodothermia	0.05958	0.02604
Clostridia	0.02233	0.01767
Bacteroidia	0.01469	0.00605
others	0.01101	0.02074
**Family**		
Bacillaceae	0.12515	0.21651
Rhizobiaceae	0.31688	0.01229
Burkholderiaceae	0.11497	0.07557
Enterobacteriaceae	0.00489	0.13238
Streptococcaceae	0.01468	0.09402
Corynebacteriaceae	0.03586	0.07466
Nocardiaceae	0.02995	0.06820
Balneolaceae	0.05958	0.02604
Mycoplasmataceae	0.07723	0.00089
Micrococcaceae	0.01642	0.05099
Pseudonocardiaceae	0.01652	0.03012
Salinisphaeraceae	0.01732	0.02544
Nocardioidaceae	0.00867	0.02939
Actinobacteriaceae	0.00861	0.01666
Others	0.13247	0.12946
**Genus**		
Bacillus	0.12515	0.21653
Bartonella	0.31187	0.00866
Burkholderia	0.11479	0.07553
Cronobacter	0.00161	0.11616
Corynebacteria	0.03586	0.07466
Lactococcus	0.01255	0.09375
Nocardia	0.02922	0.06781
Mycoplasma	0.07723	0.00089
Aliifodinibius	0.05546	0.01971

Micrococcus	0.01642	0.05099
Pseudonocardia	0.01591	0.03003
Salinisphaera	0.01732	0.02545
Aeromicrobium	0.00625	0.01961
Actinobacteria	0.00861	0.01666
Others	0.15578	0.17151

Suppl. Table S4. Alpha diversity analysis of the guts and lungs microbiota in pregnant bats.

Sample ID	Reads	Richness	Chao1	Shannon
Preg.L.1	106565	239	239	1.4
Preg.L.2	81905	376	376	4.2
Preg.L.3	53700	217	217	3.44
Preg.L.4	87266	325	325	4.41
Preg.L.5	101780	429	429	5.37
Preg.L.6	68783	361	361	5.03
Preg.L.7	97455	525	525	5.37
Preg.L.8	85285	416	416	3.54
Preg.L.9	103302	470	470	4.97
Preg.G.1	111952	288	288	0.871
Preg.G.2	93560	482	482	5.65
Preg.G.3	89065	316	316	5.16
Preg.G.4	102352	459	459	5.65
Preg.G.5	106664	649	649	5.03
Preg.G.6	105716	383	383	3.61
Preg.G.7	106594	314	314	2.23
Preg.G.8	99120	344	344	5.43
Preg.G.9	94039	321	321	5.22

Suppl. Table S5. Beta diversity analysis of the guts and lungs microbiota in pregnant bats.

Unweighted UniFrac PCoA abundance		
Samples	PCoA1 (21.2%]	PCoA2 (11.2%)
Preg.L.1	-0.028983	0.311585
Preg.L.2	-0.244176	-0.062650
Preg.L.3	-0.326985	0.150836
Preg.L.4	-0.275127	0.001445
Preg.L.5	0.125608	-0.229179
Preg.L.6	-0.179010	0.000795
Preg.L.7	-0.040951	-0.316976
Preg.L.8	-0.277243	-0.077657
Preg.L.9	0.213567	-0.081448
Preg.G.1	0.111688	0.223554
Preg.G.2	-0.290098	-0.086146

Preg.G.3	0.065475	0.135660
Preg.G.4	0.209761	-0.072271
Preg.G.5	0.249376	-0.085354
Preg.G.6	0.231865	0.014400
Preg.G.7	0.183393	0.125354
Preg.G.8	0.142404	0.024779
Preg.G.9	0.129436	0.023272
**Weighted UniFrac PCoA abundance**		
Samples	PCoA1 (34.1%]	PCoA2 (28.8%)
Preg.L.1	-0.002398	-0.297961
Preg.L.2	0.102232	-0.167565
Preg.L.3	0.401545	0.093506
Preg.L.4	0.286798	0.158694
Preg.L.5	-0.077664	0.128137
Preg.L.6	-0.005685	0.088239
Preg.L.7	-0.076785	0.126779
Preg.L.8	0.024991	-0.201377
Preg.L.9	-0.056243	0.110217
Preg.G.1	0.065718	-0.176800
Preg.G.2	-0.007058	-0.027159
Preg.G.3	0.019183	0.007702
Preg.G.4	-0.141459	0.121436
Preg.G.5	-0.157757	0.039971
Preg.G.6	-0.150470	0.012354
Preg.G.7	-0.069124	-0.097664
Preg.G.8	-0.040160	0.053931
Preg.G.9	-0.115665	0.027560

Suppl. Table S6. Bray Curtis heatmap values, exhibiting dissimilarity among the samples.

Samples	Gut-1	Gut-2	Gut-3	Gut-4	Gut-5	Gut-6	Gut-7	Gut-8	Gut-9	Lung-1	Lung-2	Lung-3	Lung-.4	Lung-5	Lung-6	Lung-7	Lung-8	Lung-9
Gut-1	0	0.9794	0.9855	0.9857	0.9592	0.9564	0.9348	0.9805	0.9833	0.9587	0.9701	0.9738	0.9719	0.9672	0.9761	0.9775	0.9601	0.9625
Gut-2	0.9794	0	0.4019	0.6364	0.6789	0.7972	0.9264	0.5646	0.4378	0.9753	0.7100	0.6975	0.6389	0.7248	0.5670	0.6702	0.8549	0.7344
Gut-3	0.9855	0.4019	0	0.6455	0.6993	0.7693	0.9221	0.5281	0.4752	0.9913	0.7191	0.6059	0.5805	0.7362	0.5286	0.6944	0.8757	0.7289
Gut-4	0.9857	0.6364	0.6455	0	0.4344	0.5025	0.9235	0.4843	0.3872	0.9916	0.8968	0.9329	0.7867	0.4617	0.5199	0.4176	0.8891	0.4165
Gut-5	0.9592	0.6789	0.6993	0.4344	0	0.3143	0.8767	0.4818	0.4628	0.9728	0.9010	0.9433	0.8032	0.5420	0.6111	0.5340	0.8891	0.4218
Gut-6	0.9564	0.7972	0.7693	0.5025	0.3143	0	0.8538	0.5605	0.5289	0.9730	0.9057	0.9430	0.8000	0.5766	0.5558	0.5693	0.8881	0.4360
Gut-7	0.9348	0.9264	0.9221	0.9235	0.8767	0.8538	0	0.8741	0.9183	0.9711	0.9377	0.9527	0.9077	0.8676	0.9152	0.8982	0.9161	0.8856
Gut-8	0.9805	0.5646	0.5281	0.4843	0.4818	0.5605	0.8741	0	0.4995	0.9863	0.7259	0.6730	0.5859	0.5879	0.4565	0.5816	0.8589	0.5577
Gut-9	0.9833	0.4378	0.4752	0.3872	0.4628	0.5289	0.9183	0.4995	0	0.9888	0.8023	0.8361	0.7388	0.5455	0.5199	0.4900	0.8593	0.5040
Lung-1	0.9587	0.9753	0.9913	0.9916	0.9728	0.9730	0.9711	0.9863	0.9888	0	0.4879	0.9804	0.9751	0.8300	0.9809	0.8395	0.2366	0.8447
Lung-2	0.9701	0.7100	0.7191	0.8968	0.9010	0.9057	0.9377	0.7259	0.8023	0.4879	0	0.6338	0.6451	0.7790	0.6921	0.7547	0.3780	0.7342
Lung-3	0.9738	0.6975	0.6059	0.9329	0.9433	0.9430	0.9527	0.6730	0.8361	0.9804	0.6338	0	0.2622	0.8752	0.6361	0.8546	0.8341	0.8464
Lung-4	0.9719	0.6389	0.5805	0.7867	0.8032	0.8000	0.9077	0.5859	0.7388	0.9751	0.6451	0.2622	0	0.7454	0.5130	0.7197	0.8168	0.7047
Lung-5	0.9672	0.7248	0.7362	0.4617	0.5420	0.5766	0.8676	0.5879	0.5455	0.8300	0.7790	0.8752	0.7454	0	0.5636	0.1082	0.7622	0.3536

Lung-6	0.9761	0.5670	0.5286	0.5199	0.6111	0.5558	0.9152	0.4565	0.5199	0.9809	0.6921	0.6361	0.5130	0.5636	0	0.5202	0.8398	0.5777
Lung-7	0.9775	0.6702	0.6944	0.4176	0.5340	0.5693	0.8982	0.5816	0.4900	0.8395	0.7547	0.8546	0.7197	0.1082	0.5202	0	0.7373	0.3460
Lung-8	0.9601	0.8549	0.8757	0.8891	0.8891	0.8881	0.9161	0.8589	0.8593	0.2366	0.3780	0.8341	0.8168	0.7622	0.8398	0.7373	0	0.7782
Lung-9	0.9625	0.7344	0.7289	0.4165	0.4218	0.4360	0.8856	0.5577	0.5040	0.8447	0.7342	0.8464	0.7047	0.3536	0.5777	0.3460	0.7782	0

Suppl. Table S7. Euclidean heatmap values, exhibiting dissimilarity among the samples.

Samples	Gut-1	Gut-2	Gut-3	Gut-4	Gut-5	Gut-6	Gut-7	Gut-8	Gut-9	Lung-1	Lung-2	Lung-3	Lung-4	Lung-5	Lung-6	Lung-7	Lung-8	Lung-9
Gut-1	0	1.4104	1.4109	1.4093	1.4016	1.4052	1.3978	1.4090	1.4092	1.3977	1.4012	1.4123	1.4112	1.3919	1.4095	1.4046	1.4002	1.3947
Gut-2	1.4104	0	0.4372	1.0564	1.1309	1.2121	1.3918	0.8827	0.7330	1.3810	1.1850	1.1124	1.0840	1.1647	0.8702	1.0992	1.3243	1.1447
Gut-3	1.4109	0.4372	0	1.0726	1.1289	1.1785	1.3881	0.7120	0.8069	1.4115	1.1357	0.9495	0.9323	1.1808	0.7317	1.1272	1.3400	1.1539
Gut-4	1.4093	1.0564	1.0726	0	0.4567	0.5087	1.3495	0.6922	0.5183	1.4099	1.3632	1.3684	1.1990	0.5981	0.7354	0.5501	1.3768	0.5509
Gut-5	1.4016	1.1309	1.1289	0.4567	0	0.3197	1.3396	0.6520	0.5709	1.3940	1.3640	1.3873	1.2479	0.7603	0.8606	0.7204	1.3682	0.5251
Gut-6	1.4052	1.2121	1.1785	0.5087	0.3197	0	1.3412	0.7212	0.6149	1.4009	1.3742	1.3947	1.2514	0.7813	0.8087	0.7477	1.3765	0.5191
Gut-7	1.3978	1.3918	1.3881	1.3495	1.3396	1.3412	0	1.2674	1.3543	1.4064	1.4040	1.4088	1.3950	1.2854	1.3653	1.3179	1.4015	1.3403
Gut-8	1.4090	0.8827	0.7120	0.6922	0.6520	0.7212	1.2674	0	0.6558	1.4009	1.1916	1.0790	0.9720	0.8940	0.5947	0.8616	1.3367	0.7899
Gut-9	1.4092	0.7330	0.8069	0.5183	0.5709	0.6149	1.3543	0.6558	0	1.4070	1.3067	1.2914	1.1639	0.8247	0.7300	0.7458	1.3654	0.6588
Lung-1	1.3977	1.3810	1.4115	1.4099	1.3940	1.4009	1.4064	1.4009	1.4070	0	0.5099	1.4048	1.3930	1.0846	1.3943	1.1051	0.1154	1.1702
Lung-2	1.4012	1.1850	1.1357	1.3632	1.3640	1.3742	1.4040	1.1916	1.3067	0.5099	0	1.1701	1.1799	1.0818	1.1887	1.0874	0.4207	1.1262
Lung-3	1.4123	1.1124	0.9495	1.3684	1.3873	1.3947	1.4088	1.0790	1.2914	1.4048	1.1701	0	0.2664	1.2800	1.0823	1.2710	1.3533	1.2525
Lung-4	1.4112	1.0840	0.9323	1.1990	1.2479	1.2514	1.3950	0.9720	1.1639	1.3930	1.1799	0.2664	0	1.1022	0.9510	1.0910	1.3391	1.0896
Lung-5	1.3919	1.1647	1.1808	0.5981	0.7603	0.7813	1.2854	0.8940	0.8247	1.0846	1.0818	1.2800	1.1022	0	0.8460	0.1180	1.0504	0.5006
Lung-6	1.4095	0.8702	0.7317	0.7354	0.8606	0.8087	1.3653	0.5947	0.7300	1.3943	1.1887	1.0823	0.9510	0.8460	0	0.8015	1.3289	0.8605
Lung-7	1.4046	1.0992	1.1272	0.5501	0.7205	0.7477	1.3179	0.8616	0.7458	1.1051	1.0874	1.2710	1.0910	0.1180	0.8015	0	1.0676	0.4666
Lung-8	1.4002	1.3243	1.3400	1.3768	1.3682	1.3765	1.4015	1.3367	1.3654	0.1154	0.4207	1.3533	1.3391	1.0504	1.3289	1.0676	0	1.1402
Lung-9	1.3947	1.1447	1.1539	0.5509	0.5251	0.5191	1.3403	0.7899	0.6588	1.1702	1.1262	1.2525	1.0896	0.5006	0.8605	0.4666	1.1402	0

Suppl. Table S8. Differential abundance taxonomic biomarkers in the guts and lungs of pregnant bats.

Biomarker ID	LDA	*p* value	Group
o_Saccharimonadales	3.002	0.001	Gut
o_Saccharimonadales	3.003	0.001	Gut
o_Saccharimonadales	3.003	0.001	Gut
o_Saccharimonadales	3.130	0.001	Gut
c_Saccharimonadia	3.130	0.001	Gut
p_Patescibacteria	3.137	0.001	Gut
f_Micrococcaceae	2.861	0.003	Gut
f_Micrococcaceae	2.861	0.003	Gut
g_Virgibacillus	2.869	0.005	Gut
g_Virgibacillus	2.845	0.005	Gut
g_Aeromicrobium	3.838	0.012	Gut
o_Micrococcales	4.255	0.012	Gut
g_Aeromicrobium	3.838	0.012	Gut
o_Micrococcales	4.255	0.012	Gut

o_Micrococcales	4.255	0.012	Gut
g_Leuconostoc	4.004	0.012	Gut
g_Leuconostoc	4.004	0.012	Gut
o_Propionibacteriales	4.034	0.015	Gut
f_Nocardioidaceae	4.023	0.015	Gut
o_Micrococcales	4.332	0.019	Gut
c_Acidimicrobiia	2.989	0.019	Gut
f_Saccharimonadaceae	2.689	0.024	Gut
p_Nitrospirota	2.687	0.024	Gut
c_Nitrospiria	2.687	0.024	Gut
f_Nitrospiraceae	2.687	0.024	Gut
g_Nitrospira	2.688	0.024	Gut
o_Nitrospirales	2.688	0.024	Gut
f_Erysipelotrichaceae	3.109	0.026	Gut
g_Turicibacter	3.231	0.029	Gut
g_Scopulibacillus	2.828	0.029	Gut
g_Turicibacter	3.231	0.029	Gut
f_Sporolactobacillaceae	2.828	0.029	Gut
f_Fusibacteraceae	3.270	0.029	Gut
s_Scopulibacillus daqui	2.828	0.029	Gut
g_Fusibacter	3.270	0.029	Gut
s_Fusibacter_fontis	3.322	0.029	Gut
f_Rhizobiaceae	2.737	0.030	Gut
f_Rhizobiaceae	2.737	0.030	Gut
f_Diplorickettsiaceae	2.787	0.030	Gut
g_Rickettsiella	2.790	0.030	Gut
o_Diplorickettsiales	2.787	0.030	Gut
f_Nocardioidaceae	3.224	0.031	Gut
f_Nocardioidaceae	3.224	0.031	Gut
g_Candidatus Rhabdochlamydia	3.256	0.031	Gut
o_Lactobacillales	3.250	0.040	Gut
o_Lactobacillales	3.250	0.040	Gut
o_Lactobacillales	3.239	0.040	Gut
g_Devosia	2.549	0.041	Gut
f_Rhodobacteraceae	3.059	0.047	Gut
o_Rhodobacterales	3.059	0.047	Gut
c_Actinobacteria	4.941	0.047	Gut
f_Nakamurellaceae	3.553	0.047	Gut
p_Actinobacteriota	4.947	0.047	Gut
g_Nakamurella	3.553	0.047	Gut
o_Frankiales	3.554	0.047	Gut
f_Rhizobiaceae	5.107	0.002	Lung
c_Alphaproteobacteria	5.112	0.002	Lung
g_Bartonella	5.103	0.002	Lung

o_Rhizobiales	5.113	0.002	Lung
g_Bartonella	5.103	0.002	Lung
s_Mycoplasma coccoides	4.632	0.004	Lung
g_Aquabacterium	2.857	0.004	Lung
s_Methylobacterium komagatae	3.081	0.006	Lung
g_Marinomonas	3.565	0.008	Lung
g_Marinomonas	3.565	0.008	Lung
f_Marinomonadaceae	3.564	0.008	Lung
o_Mycoplasmatales	4.636	0.009	Lung
g_Mycoplasma	4.636	0.009	Lung
f_Mycoplasmataceae	4.636	0.009	Lung
o_Oscillospirales	2.594	0.010	Lung
o_Oscillospirales	2.593	0.010	Lung
f_Peptostreptococcaceae	3.619	0.010	Lung
f_Peptostreptococcaceae	3.619	0.010	Lung
g_Streptococcus	2.977	0.012	Lung
f_Comamonadaceae	2.986	0.012	Lung
g_Streptococcus	2.962	0.012	Lung
g_Anoxybacillus	2.619	0.012	Lung
f_Methylopilaceae	3.354	0.012	Lung
g_Caldibacillus	3.197	0.012	Lung
g_Anoxybacillus	2.619	0.012	Lung
f_Methylopilaceae	3.354	0.012	Lung
f_Methylopilaceae	3.354	0.012	Lung
s_Caldibacillus debilis	3.197	0.012	Lung
g_Comamonas	2.770	0.014	Lung
g_Comamonas	2.770	0.014	Lung
p_Proteobacteria	3.727	0.018	Lung
p_Proteobacteria	3.730	0.018	Lung
p_Proteobacteria	3.727	0.018	Lung
p_Proteobacteria	3.729	0.018	Lung
p_Proteobacteria	3.730	0.018	Lung
f_Vibrionaceae	3.539	0.019	Lung
g_Vibrio	3.539	0.019	Lung
o_Vibrionales	3.539	0.019	Lung
g_Vibrio	3.539	0.019	Lung
f_Deinococcaceae	2.926	0.024	Lung
g_Deinococcus	2.926	0.024	Lung
g_Pseudoalteromonas	3.087	0.024	Lung
o_Alteromonadales	3.098	0.024	Lung
f_Pseudoalteromonadaceae	3.087	0.024	Lung
g_Pseudoalteromonas	3.087	0.024	Lung
f_Oxalobacteraceae	2.579	0.027	Lung
f_Oxalobacteraceae	2.544	0.031	Lung

f_Oxalobacteraceae	2.546	0.031	Lung
g_Novosphingobium	3.001	0.031	Lung
s_Lysobacter mobilis	2.862	0.031	Lung
g_Novosphingobium	2.998	0.031	Lung
g_Lysobacter	2.861	0.031	Lung
g_Aquabacterium	2.832	0.031	Lung
g_Devosia	2.776	0.033	Lung
o_Oscillospirales	2.613	0.033	Lung
f_Devosiaceae	2.790	0.033	Lung
f_Pasteurellaceae	3.103	0.038	Lung
f_Pasteurellaceae	3.103	0.038	Lung
f_Pasteurellaceae	3.103	0.038	Lung
o_Pasteurellales	3.103	0.038	Lung
o_Kineosporiales	2.715	0.039	Lung
f_Kineosporiaceae	2.715	0.039	Lung
s_Aureimonas altamirensis	2.538	0.040	Lung

Suppl. Table S9. RandomForest-based taxonomic feature importance in the guts and lung microbiota of pregnant bats.

Taxon	Mean Decrease Accuracy
Patescibacteria	9.7257
Actinobacteriota	5.2668
Proteobacteria	3.6678
Bacteroidota	3.3302
Nitrospirota	3.2132
Verrucomicrobiota	2.4668
Firmicutes	1.1036
Synergistota	1.0010
Bdellovibrionota	1.0010
Spirochaetota	0.7668
Fibrobacterota	0.6520
Deinococcota	0.3560
Unclassified bacteria	0.0551
Zixibacteria	0
Armatimonadota	0
**Taxon**	**Mean Decrease Gini**
Patescibacteria	1.7031
Actinobacteriota	0.8685
Proteobacteria	0.5862
Verrucomicrobiota	0.4974
Bacteroidota	0.4952
Deinococcota	0.4494
Nitrospirota	0.4175

Firmicutes	0.4048
Desulfobacterota	0.3902
Chloroflexi	0.3383
Dependentiae	0.3303
Unclassified bacteria	0.2620
Acidobacteriota	0.2386
Planctomycetota	0.2273
Fibrobacterota	0.2030
